# Two new species of *Allocheilos* (Gesneriaceae) from the karst regions in Yunnan, China

**DOI:** 10.3897/phytokeys.157.32729

**Published:** 2020-08-26

**Authors:** Wen-Hong Chen, Shi-Wei Guo, Jian-Yong Wu, Li Chen, Yu-Min Shui

**Affiliations:** 1 CAS Key Laboratory for Plant Diversity and Biogeography of East Asia, Kunming Institute of Botany, Chinese Academy of Sciences, 132 Lanhei Road, Kunming 650201, Yunnan, China; 2 Nanjing Institute of Environmental Sciences, Ministry of Ecology and Environment, Nanjing 210042, China; 3 School of Life Sciences, Yunnan University, Kunming 650091, Yunnan, China; 4 University of the Chinese Academy of Sciences, Beijing 100049, China; 5 Karst Conservation Initiative of Yunnan, Kunming 650201, Yunnan, China

**Keywords:** *
Allocheilos
*, corolla, habitat, isolated distribution, staminodes

## Abstract

*Allocheilos* W.T.Wang in Gesneriaceae was described in 1983 and is characterized by its 4-lobed adaxial lip and undivided abaxial lip with acute apex. The genus is endemic to the karst regions in southwestern China and is classified as endangered due to habitat loss. During surveys of the karst areas in Yunnan of southwestern China in 2017, we collected two unknown species of the genus and later confirmed their novelty to science based on the detailed observation of their morphological characteristics, viz. *A. maguanensis* W.H.Chen & Y.M.Shui and *A. rubroglandulosus* W.H.Chen & Y.M.Shui. Their relationships with the similar species and provisional conservation status are discussed.

## Introduction

**Figure 1.**
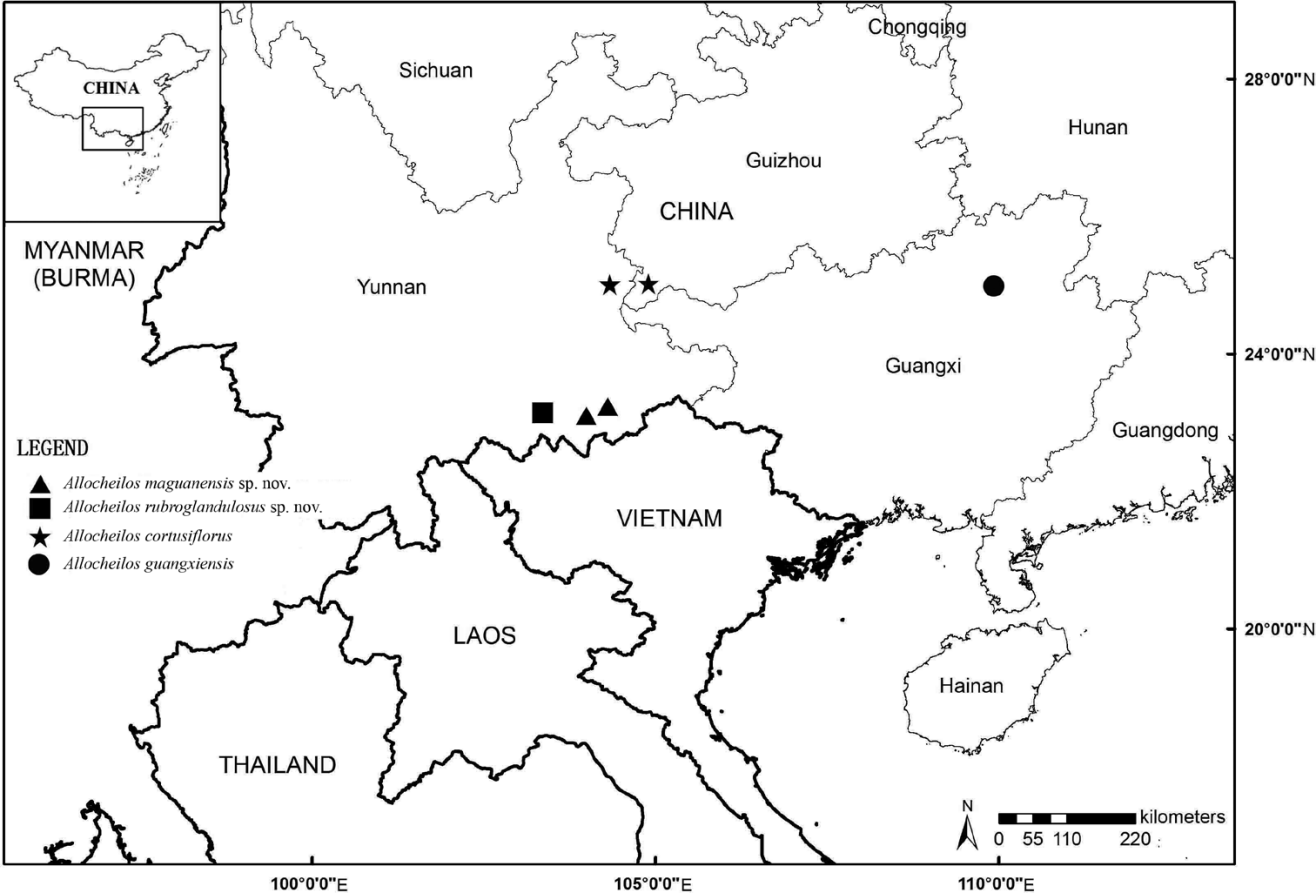
Geographical distribution of the genus *Allocheilos* W.T.Wang of Gesneriaceae in southwestern China. *Allocheilos maguanensis* W.H.Chen & Y.M.Shui, sp. nov. (▲), *A. rubroglandulosus* W.H.Chen & Y.M.Shui sp. nov. (■), *A. cortusiflorus* W.T.Wang (★), *A. guangxiensis* H.Q.Wen, Y.G.Wei & S.H.Zhong (●).

The genus *Allocheilos* W.T.Wang in Gesneriaceae is endemic to the karst regions of China and restricted to a shady habitat of limestone hills. The genus shows a stable flower morphology and is characterized by its 4-lobed adaxial corolla lip and undivided abaxial lip (Wang 1983; Wang et al. 1998), except *Oreocharis mileensis* (W.T.Wang) Mich.Möller & A.Weber, *Petrocodon coccineus* (C.Y.Wu ex H.W.Li) Yin Z.Wang and *Pet. viridescens* W.H.Chen & Y.M.Shui (Li 1982; Weitzman et al.1998; Möller et al. 2011; Wang et al. 2011; Chen et al. 2014a, b). However, the latter three exceptions are characterized by the long and narrow corolla tubes, which is different from morphology of flowers in *Allocheilos*. So far, there are only two recorded species recognized in *Allocheilos*, which show only weak morphological differences (Wei et al. 2010). They grow on shady rock surfaces of limestone hills in the Dian-Qian-Gui region, *viz.* Yunnan, Guizhou and Guangxi region in China (Fig. 1; Fang et al. 1995), and respectively have about 36 individuals of *Allocheilos cortusiflorus* W.T.Wang (30 in Xingyi, Guizhou and 6 in Luoping) and 40 individuals of *A. guangxiensis* H.Q.Wen, Y.G.Wei & S.H.Zhong in the field and thus acknowledged as critically endangered (Wang 1983; Wei et al. 2000; Wei et al. 2010).

**Figure 2.**
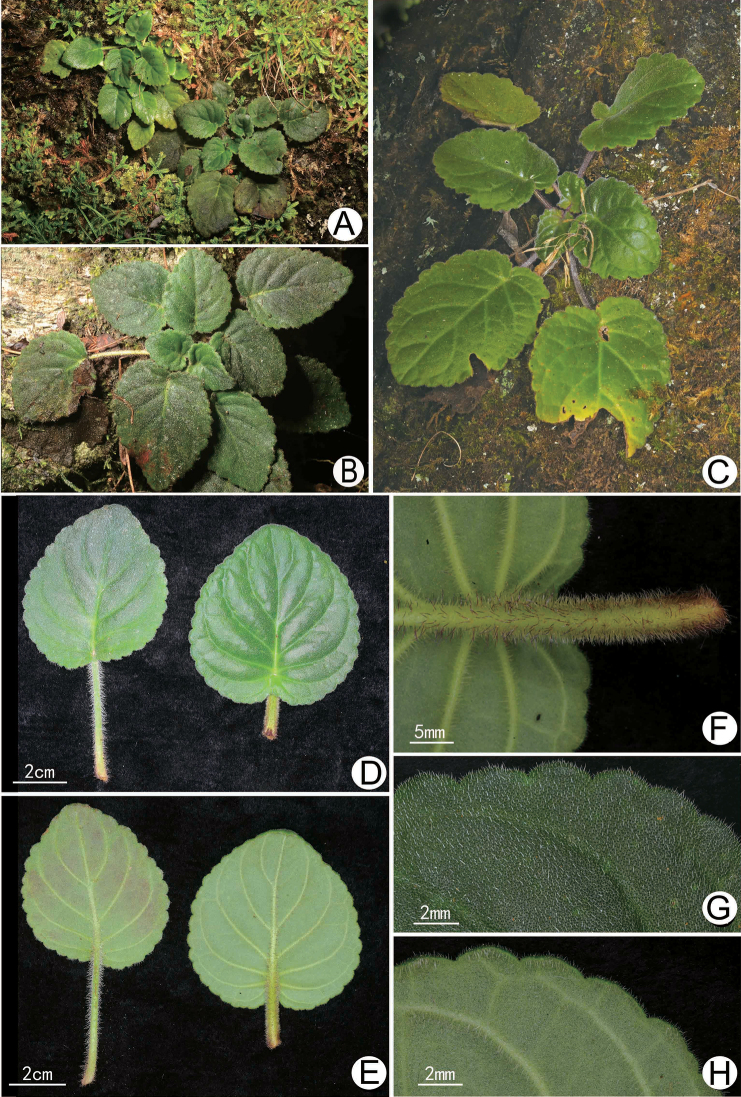
Vegetative morphology of *Allocheilos maguanensis* W.H. Chen & Y.M. Shui, sp. nov. (**A, B, D–H** from *Y.M. Shui, W.H. Chen, S.W. Guo, H.H. Xi et al. B2018-495***C** from *Y.M. Shui, S.W. Guo et al. B2017-1343*) **A–C** habit and plants **D** adaxial surface of leaves **E** abaxial surface of leaves **F** close-up of petiole **G** indumenti on the adaxial surface of leaves **H** indumenti on the abaxial surface of leaves.

**Figure 3.**
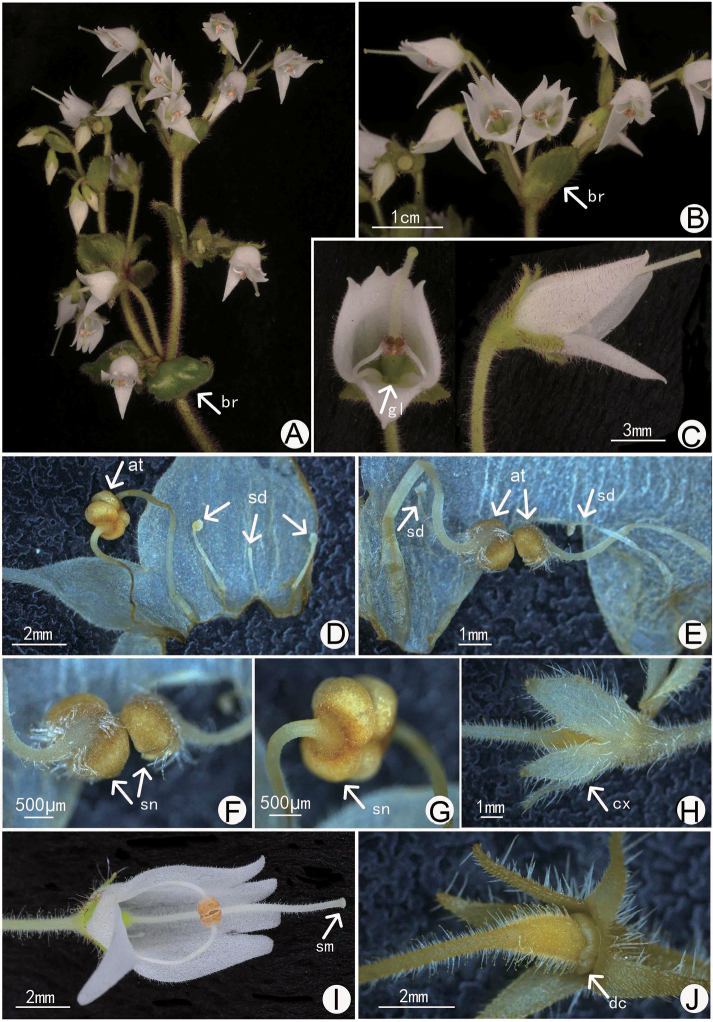
Flower morphology of *Allocheilos maguanensis* W.H. Chen & Y.M. Shui, sp. nov. (**A–C, E, F, H** from *Y.M. Shui, S.W. Guo et al. B2017-1343***D, G, I, J** from *Y.M. Shui, W.H. Chen, S.W. Guo, H.H. Xi et al. B2018-495*) **A, B** inflorescence showing the wide bracts **C** face and lateral view of flowers **D, E** opened corolla showing stamens and staminodes **F, G** close-up of stamens coherent laterally **H** calyx showing ovate segments with beaked apex **I** face view of flower showing the white staminode inside **J** disk at the base of the ovary. Notes: **at** anther, **br** bract, **cx** calyx lobe, **dc** disc, **fm** filament, **gl** glandular, **sd** staminode, **sm** stigma.

**Figure 4.**
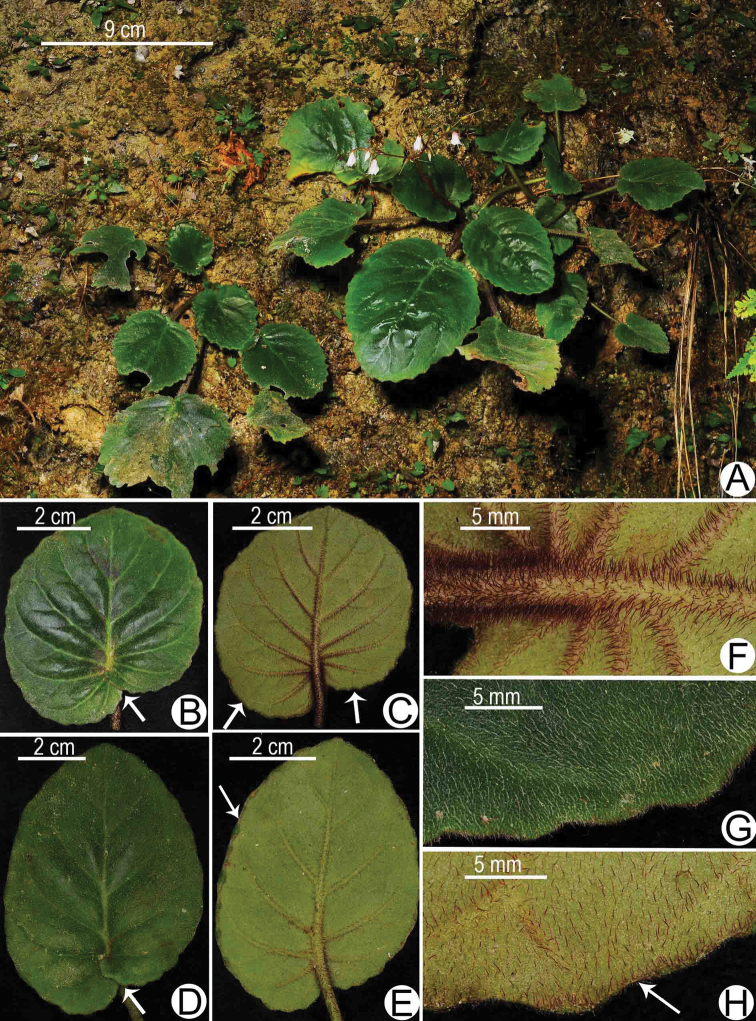
Vegetative morphology of *Allocheilos rubroglandulosus* W.H. Chen & Y.M. Shui, sp.nov. (From holotype, photographed by S. W. Guo and Y. M. Shui) **A** habitat and plants **B** adaxial surface of the round leave, arrow indicating the overlapped leaf blade at base **C** abaxial surface of the round leave, arrows indicating the reflected margin **D** adaxial surface of the broadly ovate leave with overlapped basal lobes of the leave annotated by arrow **E** abaxial surface of the broadly ovate leave, arrow indicating the reflected margin **F** hairs of petiole and main nerves on the abaxial surface of the round leaves **G** margin and hairs on the adaxial surface of the round leave **H** margin and hairs on the abaxial surface of the round leaf, arrow indicating the reflected margin.

**Figure 5.**
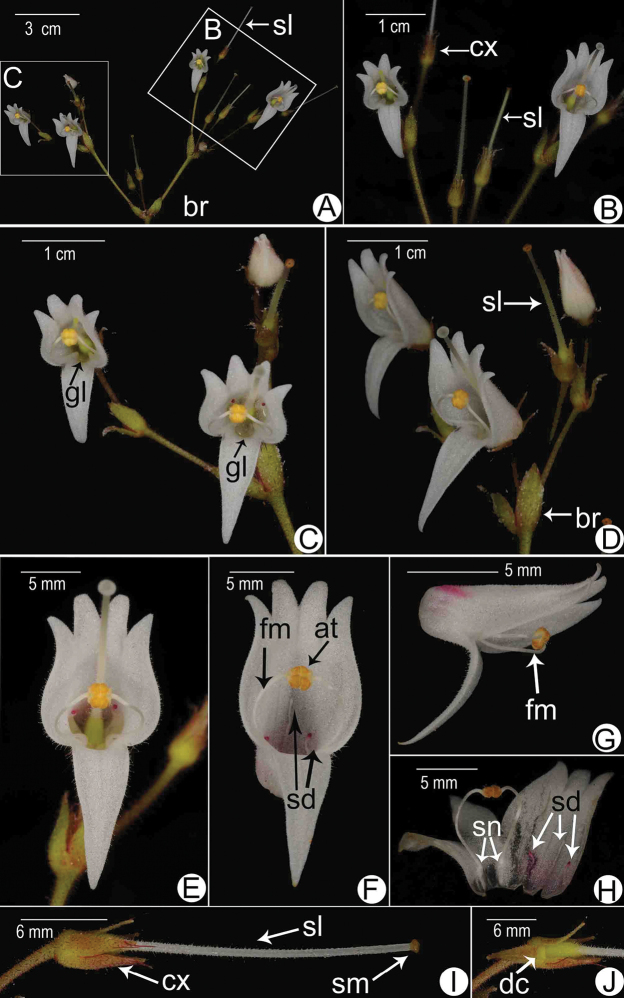
Flower morphology of *Allocheilos rubroglandulosus* W.H. Chen & Y.M. Shui, sp. nov. (From holotype, photographed by S. W. Guo and Y. M. Shui) **A** inflorescence **B** right part of the inflorescence **C** left part of the inflorescence **D** lateral view of the leaf part of the inflorescence **E** face view of the flower **F** face view indicating 3 staminodes, middle white and lateral 2 red on the top **G** lateral view of flower indicating stamens **H** opened corolla indicating the stamens and staminodes and the swollen nodes at the base of filaments. Notes: **at** anther, **br** bract; **cx** calyx lobe, **dc** disc, **fm** filament, **gl** glandular, **sd** staminode, **sl** style, **sm** stigma, **sn** swollen nodes at base of filament.

During surveys in the karst regions in Yunnan in 2016 and 2017 we encountered two unknown species of *Allocheilos*. At first glance, they look like the species of *Petrocodon* without flowers in the adjacent region of southeastern Yunnan, such as *Pet. viridescens* W.H.Chen & Y.M.Shui (Chen et al. 2014a). However, they are characterized by white 4-lobed adaxial corolla lips and short corolla tubes, showing that they are a member of *Allocheilos* (Figs 2–5). Based on the examination of relevant publications and specimens of *Allocheilos* from E, IBK, K and P, we found that they are more similar to *A. cortusiflorus* with short ovary and long style than *A. guangxiensis* with long ovary and short style (Pellegrin 1930; Wang et al. 1990; Li and Wang 2005; Wei et al. 2010). Furthermore, after the detailed morphological and geographical comparison, we realized that they represent two different species (Table 1). Firstly, there are obvious morphological differences in bracts and staminodes. Secondly, there is a geographical isolation between them, which are respectively distributed in Maguan county and Mengzi county, separated by the Naxi River, SE Yunnan, China (Fig. 1). Thirdly, there are obvious different habitats between them, one of which grows near the summit of the limestone hills and another in the deep limestone sinkholes. So here we described the two species of *Allocheilos* unknown to science.

## Materials and methods

The materials are from type specimens and other specimens kept in the herbarium of Kunming Institute of Botany, Chinese Academy of Sciences (KUN). Photographs of the habitat, plants, leaf blade and flowers were taken in the field and greenhouse with camera (Nikon D700, Tokyo, Japan), and detailed morphological characteristics were observed and photographed with stereomicroscope (Leica S8 APO, Serial number 5683759, Shanghai, China). Table 1 is provided to explain the relationship among the two new species and their similar species of *Allocheilos*.

**Table 1.** Morphological comparison of *Allocheilos maguanensis* sp. nov., *A. rubroglandulosus* sp. nov., and *A. cortusiflorus* in Gesneriaceae.

Characters	*A. maguanensis*	*A. rubroglandulosus*	*A. cortusiflorus*
Bracts	broadly ovate to rounded	elliptic	linear
Calyx lobes	elliptic, apex acute	triangular, apex awny	linear, apex acute
Corolla	glandular-villous outside	glandular-pubescence outside	glandular-villous outside
Adaxial lip of corolla	inner two lobes bigger than outer two lobes	inner two lobes bigger than outer two lobes	inner two lobes almost equal to outer two lobes
Abaxial lip of corolla	triangular, slightly reflexed, apex acute	triangular, extremely reflexed, apex acute	triangular, slightly reflexed, apex acute
Filaments	apex glabrescent	apex glabrous	apex glabrescent
Staminodes	3, major, 3–4 mm long	3, major, 3–4 mm long	2, minor, 0.4–0.5 mm long
Top of lateral staminodes	without red glands	with red glands	without red glands
Mature fruit	linear, 1.0–1.1 cm	elliptic, 0.7–0.8 cm	linear, 1.5–1.8 cm
Habitat	near the summit of the limestone hills	deep limestone sinkholes	deep limestone sinkholes or valleys

## Taxonomic treatments

### Allocheilos maguanensis

Taxon classificationPlantaeLamialesGesneriaceae

W.H. Chen & Y.M. Shui sp. nov.

FEA552FF-4F5E-56AA-80C9-78CFB049C607

urn:lsid:ipni.org:names:77211192-1

Figs 2 , 3

#### Diagnosis.

The new species is similar to *A. cortusiflorus* W.T.Wang in glandular-villous corolla on the adaxial surface and style longer than ovary, but differs in its broadly ovate bracts (*vs.* linear), calyx lobes elliptic (*vs.* linear), 11.1–11.3 mm long corolla (*vs.* 8.5–9.5 mm), inner two lobes bigger than outer two lobes (*vs.* equal between them), staminoides major 3–4 mm long (*vs.* minor 0.4–0.5 mm), and 1.0–1.1 cm longer mature fruit (*vs.* 1.5–1.8 cm).

#### Type.

China. Yunnan province, Maguan county, Bazhai town, 23°06'34"N, 104°03'18"E, on the cliffs of a limestone mountain, elev. ca. 1750 m, introduced on 7 April 2017 from the above locality to Kunming Botanic Garden (KBG), in flower on 11 July 2017 in KBG and prepared for specimens there, *Y.M. Shui, S.W. Guo et al. B2017-1343* (holotype, KUN).

Herbs perennial. Rhizome short, stem absent. Leaves basal, opposite; petiole 7–13 cm long, brown-red villous; blade herbaceous, slightly asymmetric, ovate to rounded, 7.5–10 × 7–9.5 cm, base cordate, apex obtuse, margin crenate, adaxially densely depressed white pubescent, abaxially white pubescent and brown-red villous along the nerves; venation palmate, lateral veins 5–7 on each side of midrib. Inflorescences axillary, cymes with many flowers; peduncle 11–12.5 cm, densely glandular brown-red villous; bracts 2, broadly ovate to rounded, 0.8–1 × 0.7–0.8 cm, adaxially strigose, abaxially densely villous, margin crenate, apex acute; bracteoles 2, broadly ovate, ca. 6 × 3 mm, adaxially strigose, abaxially densely villous; pedicel ca. 1–1.5 cm, glandular-villous. Calyx 5-parted to the base; segments elliptic, 4 × 1–2 mm, apex beaked, margin entire, adaxially sparsely glandular brown-red villous, abaxially densely pubescent. Corolla white, campanulate, zygomorphic, 1–1.3 cm long, ca. 0.4 cm wide at the throat, outside glandular-villous, inside glabrous, tube ca. 4 mm long; limb 2-lipped; adaxial lip ca. 9 mm long, 4-lobed to 1/3 of the lip, lobes triangular, ca. 3 mm long; abaxial lip lanceolate, undivided, ca. 9 × 3 mm. Stamens 2, adnate to the corolla ca. 2 mm above base; anthers 2, coherent laterally; filaments ca. 6 mm long, sparsely villous above middle and barbate near the anthers; staminodes 3, glabrous, adnate to the corolla tube ca. 2 mm above base. Disc ring-like, ca. 0.5 mm high. Pistil 1.5–1.8 cm long; ovary oblong, ca. 3 mm long, densely strigose and glandular strigose; style linear, ca. 1.5 cm long, densely strigose and glandular strigose; stigma 1, capitate. Capsule linear, 1.0–1.1 cm long.

#### Phenology.

Flowering is from June to July (in the greenhouse).

#### Etymology.

The epithet means the type locality of the new species.

#### Distribution and ecology.

The new species is only distributed in the type locality, SE Yunnan, SW China. It grows in the limestone evergreen broad-leaved forests near the summit of the limestone hills. The main companying plants are *Sinosenecia oldhamianus* (Maxim.) B. Nord. in Compositae, *Petrocosmea minor* Hemsl. and *Paraboea rufescens* (Franch.) Burtt in Gesneriaceae, *Scutellaria sichourensis* C.Y.Wu et H.W.Li in Labiatae, *Eria coronaria* (Lindl.) Rchb. f., and *Bulbophyllum andersonii* (Hook.f.) J.J.Sm in Orchidaceae.

#### Provisional conservation status.

Based on observations around the locality, this new species was found in two localities with two minor populations in Maguan Xian, Yunnan province, China. One population grows on limestone cliff around 10 mature individuals, and was easily disturbed by human activities. Another population grows among rocks of the limestone hills with around 20 mature individuals. Thus, further exploration should be conducted while an urgent conservational project is needed for this rare species with extremely small populations (Ma et al. 2013). So far, the species can be provisionally considered as Critically Endangered, CR: B1ab (iii) + 2ab (iii). (IUCN 2012; IUCN Standards and Petitions Subcommittee 2017).

#### Additional examined specimens.

China. Yunnan province, Maguan county, Bazhai town, 7 April 2017 introduced to KBG, *S.W. Guo et al. BZL04-012* (KUN). Maguan county, Pojiao town, 23°06'28"N, 104°18'30"E, 13 October 2017 introduced to KBG, *Y.M. Shui, W.H. Chen, S.W. Guo, H.H. Xi et al. B2018-495* (KUN). Maguan county, Pojiao town, *Y.M. Shui, W.H. Chen, S.W. Guo et al. PJL07-006* (KUN); ibid, 13 October 2017, *S.W. Guo, H.H. Xi PJL14-017* (KUN).

#### Discussion.

The new species has obvious staminodes, which are usually difficult to observe on specimens in herbaria. So after the detailed observation in the field, the diversity of staminodes will provide us with an opportunity to explore its evolutionary implications for the genus in Gesneriaceae.

### Allocheilos rubroglandulosus

Taxon classificationPlantaeLamialesGesneriaceae

W.H. Chen & Y.M. Shui sp. nov.

E8C0A97D-606A-583B-AA5B-F6A9F127E687

urn:lsid:ipni.org:names:77211201-1

Figs 4 , 5

#### Diagnosis.

The new species is similar to *A. maguanensis* W.H.Chen & Y.M.Shui with three major staminodes and style longer than ovary, but differs in its elliptic bracts (*vs.* broadly ovate to rounded), triangular calyx lobes awny at the apex (*vs.* elliptic and beaked at the apex), and lateral staminodes with red glands (*vs.* without red glands).

#### Type.

China. Yunnan province, Mengzi county, Shuitian community, 23°08'35"N, 103°21'46"E, on the cliffs of a limestone sink-hole, elev. ca. 1410 m, in flower, 6 November 2017, *Y. M. Shui et al. B2017-1287* (holotype KUN; isotype PE).

Herb perennial. Rhizome short, 6–7 mm in diam., stem absent. Leaves 8–12 in mature individuals, basal, rosette; petiole 9.4–11.6 cm long, densely brown-red villous; blade herbaceous, round or broadly ovate, 4.5–13 × 3.6–9.8 cm, base obliquely cordate, apex round or obtuse, margin shallowly crenulate, slightly reflected, adaxially densely depressed white pubescent, abaxially densely brown-red villous especially along the nerves; venation palmate, lateral veins 4–7 on each side of midrib, adaxially slightly depressed and abaxially prominent. Inflorescences axillary, 10–16-flowered cymes; peduncles 8–10 cm long, densely brown-red villous; bracts 2, opposite, elliptic, 5.6–6.4 × 2.3–2.6 mm, adaxially subglabrous, abaxially sparsely brownish hispid, margin obscurely and irregularly crenulate and ciliate above the middle, apex acute; bracteoles 2, oblong-lanceolate, ca. 5 × 2 mm, adaxially subglabrous, abaxially densely brownish hispid; pedicel 0.6–1.8 cm long, brown-red hispid. Calyx actinomorphic, 5-parted to the base, segments lanceolate or triangular, 4.5–5.2 × 2.1–2.2 mm, apex acuminate or caudate, margin entire, abaxially densely white pubescent and brown-red villous, adaxially glabrous. Corolla white or pinkish at the base, broadly campanulate, zygomorphic, ca. 1.1 cm long, ca. 0.4 cm wide at the throat, outside white glandular pubescent, inside glabrous, tube 3.2–3.5 mm long; limb 2-lipped; adaxial lip 8.8–9.2 × 8.8–9.5 mm, slightly swollen at the base, ca. 6 mm high, ca. 7 mm wide, slightly longer than abaxial lip, 4-lobed, lobes triangular, 3.1–3.3 × 1.4–1.6 mm, apex obtuse, middle lobes 2 symmetric, lateral lobes 2 oblique; abaxial lip narrowly triangular, undivided, 8.1–8.5 × 2.5–2.9 mm, apex acute. Stamens 2, adnate ca. 2 mm above base; anthers 2, coherent laterally; filaments sparsely puberulent, base slightly swollen, 7–8 mm long; staminodes 3, lateral 2 red on the top, ca. 1.5 mm long, adnate to ca. 1.5 mm above base, middle white on the top, ca. 3.1 mm long. Disc ring-like, yellow, 0.7–0.8 mm high, margin entire. Pistil 1.4–1.6 cm long; ovary oblong, 3–4 mm long, sparsely pubescent; style 7–8 mm long, sparsely pubescent; stigma 1, capitate. Dry capsule straight, elliptic, 7–8 × 2.1–2.3 mm, dehiscing loculicidally, valves 2.

#### Phenology.

Flowering is from October to November (June to July in the greenhouse).

#### Etymology.

The epithet refers to the red glands on the top of lateral staminodes, which is an indicator to lead the pollinators to find the nectar. Hitherto, the character was first observed in Gesneriaceae (Weber 2004).

#### Distribution and ecology.

The new species is only distributed in Mengzi county, Yunnan, southwestern China. It grows in deep limestone sinkholes. The adjacent habitat has been disturbed by local people for the purpose of planting corn. The main accompanying plants are *Begonia laminariae* Irmscher in Begoniaceae, *Impatiens apalophylla* J.D.Hooker in Balsaminaceae, *Pteris deltodon* Baker in Pteridiaceae, *Thalictrum ichangense* Lecoyer ex Oliver in Ranunculaceae.

#### Provisional conservation status.

Based on observations around the locality, this new species was found only in one limestone sinkhole with around 30 mature individuals on the cliffs in three smaller populations. Thus, as the above new species, its further exploration should be conducted while an urgent project is needed for this rare species with extremely small populations (Ma et al. 2013). So far, the species can be provisionally considered as Critically Endangered, CR: B1b (v) + 2b (v). (IUCN 2012; IUCN Standards and Petitions Subcommittee 2017)

#### Additional examined specimens.

China. Yunnan province, Mengzi county, Shuitian community, elev. ca. 1410 m, fruits dry, 6 November 2017, *Y. M. Shui et al. B2017-1296* (KUN); ibid, *Y. M. Shui et al. B2017-1298* (KUN).

#### Discussion.

At the background of the white flowers, two red glands at the top of staminodes are an important indicator for the pollinators to visit the plants. It is obvious that nectars are hidden at the back of the indicator (Fig. 5C).The discovery will provide us an opportunity to explore the diversity and evolution of staminodes of the genus in Gesneriaceae and the other groups endemic to the karst regions in China and the adjacent countries (Laos, Myanmar, Thailand and Vietnam).

## Supplementary Material

XML Treatment for Allocheilos maguanensis

XML Treatment for Allocheilos rubroglandulosus
